# Serum Interleukin-6 in Systemic Lupus Erythematosus: Insights into Immune Dysregulation, Disease Activity, and Clinical Manifestations

**DOI:** 10.3390/cells14191568

**Published:** 2025-10-09

**Authors:** Patricia Richter, Ciprian Rezus, Alexandra Maria Burlui, Thomas Gabriel Schreiner, Elena Rezus

**Affiliations:** 1 Department of Rheumatology and Rehabilitation, “Grigore T. Popa” University of Medicine and Pharmacy, 700115 Iasi, Romania; 2 I Rheumatology Clinic, Clinical Rehabilitation Hospital, 14 Pantelimon Halipa Street, 700661 Iasi, Romania; 3 Department of Internal Medicine, “Grigore T. Popa” University of Medicine and Pharmacy, 16 University Street, 700115 Iasi, Romania; 4 III Internal Medicine Clinic, “St. Spiridon” County Emergency Clinical Hospital, 1 Independence Boulevard, 700111 Iasi, Romania; 5 Department of Medical Specialities III, “Grigore T. Popa” University of Medicine and Pharmacy, 16 University Street, 700115 Iasi, Romania; 6 First Neurology Clinic, “N. Oblu” Clinical Emergency Hospital Iasi, 2 Ateneului Street, 700309 Iasi, Romania

**Keywords:** systemic lupus erythematosus, interleukin-6, cytokines, inflammation

## Abstract

Background: Interleukin-6 (IL-6) is a multifunctional cytokine implicated in various inflammatory and immune-mediated processes. Its involvement in systemic lupus erythematosus (SLE) has been increasingly investigated, particularly related to disease activity and tissue damage. This study aimed to quantify serum IL-6 levels in patients with SLE and assess their associations with clinical manifestations and laboratory parameters. Methods: A total of 88 patients diagnosed with SLE and 87 matched healthy controls were included. Serum IL-6 concentrations were measured by ELISA. Clinical data, SLEDAI scores, organ involvement, inflammatory markers, and autoantibody profiles were recorded. The statistical analysis involved non-parametric testing, correlation analysis, and linear regression. Results: IL-6 concentrations were higher in SLE patients than in controls (7.46 ± 6.73 vs. 5.30 ± 10.89 pg/mL). Significantly increased IL-6 levels were observed in patients with active disease (SLEDAI ≥ 6; *p* = 0.025) and renal (*p* = 0.001) involvement. Positive correlations were identified between IL-6 and ESR, creatinine, ANA, and specific autoantibodies (anti-dsDNA, SSA, and SSB). IL-6 also correlated with IL-10 (*p* = 0.010) but showed no significant association with IL-17A, TNF-α, CRP, or complement levels. Conclusions: Elevated IL-6 levels are associated with greater disease activity and specific organ involvement in SLE. These findings highlight IL-6 as a measurable indicator of immunological and clinical disease expression, supporting its relevance in disease monitoring.

## 1. Introduction

Systemic lupus erythematosus (SLE) represents the prototypical autoimmune condition marked by alternating phases of exacerbation and quiescence, with the potential to cause severe damage to multiple organ systems and tissues. The onset of the disease is related to the interaction of immune dysregulation, genetic background, the gradual loss of immune tolerance, and several environmental stimuli. These intricate relationships lead to the generation of harmful autoantibodies. Together with innate and adaptive immunological responses, the lupus-associated autoantibodies promote the accumulation of immune complexes in different tissues and organs. The multifaceted nature of SLE and its tendency to affect multiple organ systems pose significant therapeutic challenges, emphasizing the need for a thorough understanding of its immunopathogenic mechanisms to develop specific and effective treatment strategies [1,2].

Among the immunological mediators involved in SLE, interleukin-6 (IL-6) has drawn substantial attention due to its multifaceted biological functions. IL-6 is a pleiotropic pro-inflammatory cytokine produced by various cell types, including immune cells (T lymphocytes, monocytes, macrophages, dendritic cells, and B lymphocytes), but also non-immune cells (endothelial cells, astrocytes, and fibroblasts) in response to infection, tissue damage, or chronic inflammation. In SLE patients, IL-6 levels were associated with disease activity, renal involvement, and abnormal B and T cell function [3,4]. At the molecular level, IL-6 enhances B cell differentiation and autoantibody production, as well as promotes Th17 cell expansion—key immunopathological processes in SLE [5,6]. Recent experimental studies have implicated IL-6 in promoting senescence and impaired autophagy of bone marrow mesenchymal stem cells in lupus-prone mice, further supporting its contribution to SLE pathogenesis [7]. Genetic polymorphisms in the IL-6 gene, particularly the promoter variant –174 G/C, may influence IL-6 transcription and modulate susceptibility to autoimmune diseases, including SLE, although findings remain inconclusive. Another promoter single nucleotide polymorphism (SNP), –572 G/C, has also been investigated, but no significant association with SLE has been established [8,9,10,11,12,13].IL-6 mediates its effects through a receptor complex composed of the IL-6 receptor (either membrane-bound or soluble) and the signal-transducing β-subunit glycoprotein 130 (gp130, CD130). This interaction triggers downstream signaling pathways, notably JAK/STAT, MAPK/ERK, and PI3K/AKT/mTOR, regulating inflammatory gene expression, immune cell differentiation, and tissue injury [3,14,15,16,17,18].

Depending on the receptor form involved, IL-6 can act through three distinct signaling modes: classic, trans-, and cluster signaling. In classic signaling, IL-6 binds to the membrane IL-6 receptor (IL-6R), which then joins with gp130 to activate a response, mainly in hepatocytes and leukocytes [3,19,20,21]. In trans-signaling, IL-6 binds to the soluble IL-6R (sIL-6R), enabling activation of cells that express gp130 but lack membrane IL-6R, such as endothelial cells and fibroblasts [3,21]. The third mechanism, cluster signaling (or trans-presentation), occurs when activated dendritic cells present the IL-6/IL-6R complex to gp130 on target cells, initiating signaling [3,14].

According to recent research, IL-6 is constantly more elevated in SLE patients than in healthy controls, and its levels are frequently positively associated with disease activity indexes such as SLEDAI. Meta-analyses and case–control studies demonstrated that higher IL-6 concentrations are linked to more active disease, albeit the strength of this association varies across populations and study designs [22,23,24].

In addition, studies examining particular manifestations of SLE showed distinct cytokine involvement patterns. Among these, IL-6 has been consistently associated with specific clinical characteristics of SLE. For instance, in Neuropsychiatric Systemic Lupus Erythematosus (NPSLE), a subset characterized by neurological and psychiatric symptoms affecting the central nervous system, IL-6 levels have demonstrated a significant correlation with the severity of cognitive dysfunction (CD), which is the most frequent manifestation [25]. Elevated serum IL-6 levels have been observed in patients with NPSLE, and its detection in cerebrospinal fluid supports the hypothesis of localized effects of IL-6 in neuropsychiatric manifestations of SLE [26,27,28]. Although IL-6 inhibitors are well established in the treatment of other autoimmune diseases, they are not currently approved for SLE, and clinical trials investigating IL-6 blockade in this context have thus far yielded limited efficacy. Nevertheless, IL-6 remains a relevant therapeutic target, particularly in SLE patients with complement dysregulation or increased cardiovascular risk, given its association with both disease pathogenesis and related comorbidities [29,30,31].

Given the central role of IL-6 in immune regulation, inflammation, and organ damage in SLE, a deeper understanding of its contribution to SLE pathogenesis is essential. In this regard, we quantified serum IL-6 levels in SLE patients and investigated their associations with clinical manifestations, laboratory parameters, disease activity, damage scores, and therapeutic interventions, also comparing them with healthy controls. Importantly, patients with SLE from Eastern Europe remain largely underrepresented in the literature, which underscores the relevance of our work and its contribution to expanding the evidence base in this population.

## 2. Materials and Methods

### 2.1. Patients and Controls

This study included 88 patients diagnosed with SLE who were followed at the Rheumatology Clinic of the Clinical Rehabilitation Hospital Iași between July and November 2022. All patients met the classification criteria for SLE, based on the set of criteria applicable at the time of diagnosis: the 1997 revised American College of Rheumatology (ACR) criteria [32], the 2012 Systemic Lupus International Collaborating Clinics (SLICC) criteria [33], or the 2019 EULAR/ACR criteria [34]. Specifically, 35 patients were diagnosed according to the 1997 ACR criteria (diagnoses between 1985 and 2011), 25 according to the 2012 SLICC criteria (diagnoses between 2012 and 2018), and 28 according to the 2019 EULAR/ACR criteria (diagnoses between 2019 and 2022). For patients diagnosed before 1997, retrospective classification was confirmed using available clinical and immunological data. Patients were excluded if they had overlap syndromes, active SARS-CoV-2 infection, cognitive or communication difficulties, or if they declined participation. Control subjects were age-, sex-, and ethnicity-matched healthy individuals with no medical history of autoimmune diseases.

This research was conducted in compliance with the principles of the Declaration of Helsinki and received approval from the Ethics Committee of the Clinical Rehabilitation Hospital Iași (approval code: 02/19 May 2022) and the “Grigore T. Popa” University of Medicine and Pharmacy, Iași (approval code: 239/28 November 2022). All participants provided written informed consent before enrollment.

### 2.2. Clinical Assessment

Demographic and clinical data, including date of birth, gender, disease duration and activity, organ involvement, and comorbidities, were obtained from patients’ medical records.

Disease activity was assessed at the time of consultation using the Systemic Lupus Erythematosus Disease Activity Index (SLEDAI), which evaluates disease status over the past 30 days based on 24 clinical and laboratory parameters [35,36]. In our study, a SLEDAI score of 6 or higher was used to define active disease.

### 2.3. Paraclinical Assessment

Blood samples were collected during scheduled follow-up visits while patients were under stable treatment with immunosuppressive agents or DMARDs. Blood samples collected from SLE patients and controls were processed by centrifugation, and the resulting sera were promptly used for routine laboratory analyses. Hematological parameters included hemoglobin concentration, leukocyte, lymphocyte, and platelet counts. Inflammatory markers such as erythrocyte sedimentation rate (ESR) and C-reactive protein (CRP) were also assessed. Standard biochemical analyses included hepatic and renal function parameters. The immunological workup comprised antinuclear antibodies (ANA), anti-double-stranded DNA (anti-dsDNA), anti-SSA, and anti-SSB antibodies. Complement components C3 and C4 were quantified by nephelometry. ANA positivity was defined as an index value >1.2 (borderline: 1.0–1.2; negative: <1.0). Anti-dsDNA and anti-SSA/SSB positivity were defined as values >25 U/mL. Hypocomplementemia was defined as C3 and/or C4 concentrations below the reference ranges (C3: 88–252 mg/dL; C4: 13–75 mg/dL).

Serum IL-6 concentrations were determined using a commercially available ELISA kit (BioVendor Laboratorní medicína a.s., Brno, Czech Republic; RD194015200R), following the manufacturer’s instructions. The sandwich ELISA method utilizes monoclonal anti-human IL-6 antibodies for specific detection. Blood samples were obtained via standard venipuncture techniques, and serum was separated by centrifugation. To maintain cytokine stability, aliquots were stored at −80 °C until analysis. Prior to measurement, samples were thawed, vortexed, and diluted threefold (1:3) with a Dilution Buffer to optimize detection within the assay’s dynamic range.

Standards, blanks, and diluted serum samples were added to microtiter wells pre-coated with capture antibodies and incubated for 60 min at room temperature under constant shaking (300 rpm). After washing, a biotinylated anti-IL-6 antibody (100× concentrate, diluted 1:100 with Dilution Buffer) was added and incubated for another 60 min. Following a second wash, Streptavidin-HRP conjugate was added and incubated for 30 min. Then, 100 μL of substrate solution was added, and the colorimetric reaction was allowed to develop for 10 min. The reaction was terminated using a stop solution, and absorbance was read at 450 nm using a microplate reader. A standard curve was generated using recombinant IL-6 standards, ranging from 1.25 to 80 pg/mL, with unknown sample concentrations interpolated accordingly. The assay’s lower limit of detection was 0.32 pg/mL. In a reference population of healthy individuals, IL-6 levels were generally below 1.25 pg/mL, as reported by the manufacturer. Final IL-6 concentrations were calculated by multiplying the measured values by the dilution factor (×3).

### 2.4. Statistical Analysis

Statistical analyses were performed using IBM SPSS Statistics version 28.0. For the descriptive analysis, quantitative variables were reported as minimum, maximum, and mean ± standard deviation, or as median with interquartile range (IQR), as appropriate. Qualitative variables were expressed as absolute frequencies and percentages.

Depending on data distribution, comparisons between two groups were made using either the independent samples *t*-test or the Mann–Whitney U test. Correlations were assessed using Pearson’s or Spearman’s rank correlation coefficients, as appropriate. Simple linear regression models were applied to explore the predictive value of clinical variables for IL-6 concentrations. A *p*-value < 0.05 was considered statistically significant. Binary logistic regression models were constructed to assess the independent association of IL-6 with clinical and immunological outcomes (renal, hematological, articular, cutaneous, and cardiovascular involvement; ANA, anti-dsDNA, anti-SSA, and anti-SSB positivity). All models were adjusted for age, gender, and disease duration, and additionally for treatment variables (corticosteroids, hydroxychloroquine, and azathioprine). Missing data were handled by listwise deletion. Odds ratios (ORs) with 95% confidence intervals (CIs) and *p*-values were reported. Model fit was evaluated using the Omnibus test of model coefficients and Nagelkerke R^2^.

## 3. Results

### 3.1. Descriptive Analysis of SLE Group and Controls

The study population included 88 patients diagnosed with SLE (mean age: 51.17 ± 15.36 years), of whom 89.8% were female and 10.2% male.

A control group of 87 age- and sex-matched healthy individuals (mean age: 51.38 ± 15.30 years) was also included for comparison.

The demographic characteristics of SLE patients and controls are summarized in Table 1.

The median disease duration among SLE patients was 7 years. In our cohort, the most common clinical manifestation at presentation was arthralgia, affecting 70 of 88 patients (79,6%), followed by fatigue, cutaneous involvement, and sicca symptoms such as xerophthalmia or xerostomia, which were less frequently observed. Based on disease activity (SLEDAI score), 25% of the cohort exhibited clinically active disease (SLEDAI ≥ 6), while 75% presented with low or inactive disease (SLEDAI < 6).

Regarding SLE manifestations, immunological involvement was the most prevalent, observed in 86 out of 88 patients; in our study, this term refers to the presence, at any time during the disease course, of abnormalities such as ANA, anti-dsDNA, anti-Sm, complement consumption, or direct Coombs positivity. Articular involvement was present in 70 SLE patients, followed closely by hematological manifestations in 69 patients. Cutaneous manifestations were identified in 58 patients, while renal involvement was reported in 28. Neurological involvement occurred in 20 patients, and serosal manifestations, such as pleuritis or pericarditis, were documented in 15. Mucosal involvement was found in 13 patients, whereas pulmonary and cardiovascular manifestations, excluding cases of serositis, were less frequent, being reported in 5 and 3 patients, respectively.

Hydroxychloroquine was the most frequently prescribed treatment (79/88, 89.8%), followed by azathioprine (30/88, 34.1%) and methotrexate (7/88, 8.0%). Corticosteroid therapy was administered in 33/88 patients (37.5%).

### 3.2. IL-6 Levels in SLE Patients Versus Healthy Controls

Serum IL-6 levels were higher in SLE patients (mean 7.46 ± 6.73 pg/mL) compared to healthy controls (mean 5.30 ± 10.89 pg/mL), as presented in Table 2. However, this difference did not reach statistical significance (*p* = 0.117, independent samples *t*-test).

### 3.3. IL-6 Levels and Age in SLE Patients

**Figure 1:**
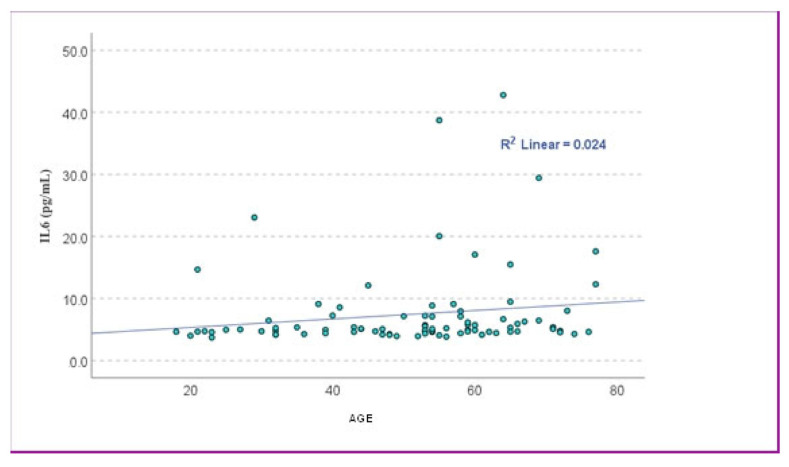
The scatter plot showed the relationship between age and IL-6 levels. The data points were widely scattered, with a very low coefficient of determination (R^2^ = 0.024), suggesting that age explained only about 2.4% of the variance in IL-6 levels.

Spearman’s correlation revealed a statistically significant but weak positive association between age and IL-6 levels (r = 0.234, *p* = 0.028). In contrast, Pearson’s correlation showed a similar positive trend (r = 0.156), though without reaching statistical significance (*p* = 0.148). To further explore this relationship, a simple linear regression model was applied using age as the independent variable and IL-6 concentration as the dependent variable. The model did not reach statistical significance (*p* = 0.148), and the regression coefficient indicated an estimated increase of 0.068 pg/mL in IL-6 for each additional year of age (β = 0.156, 95% CI: –0.025 to 0.161), but the wide confidence interval indicates limited clinical relevance (Figure 1).

### 3.4. IL-6 Levels and Gender in SLE Patients

**Figure 2:**
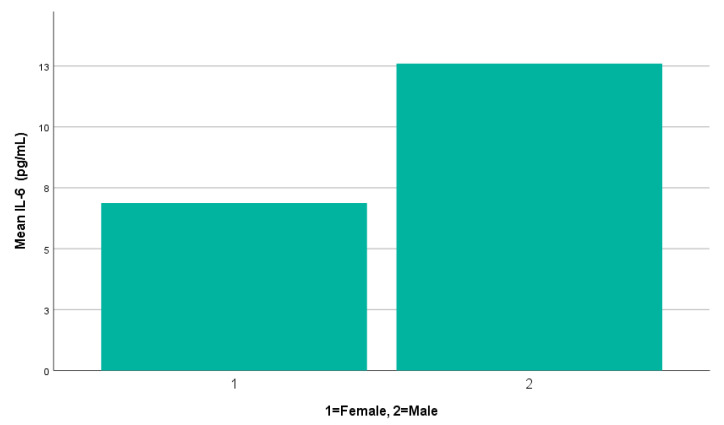
A simple bar chart illustrating mean serum IL-6 levels by gender.

Mean serum IL-6 concentrations were elevated in male patients (*n* = 9, 12.60 ± 11.51 pg/mL) compared to females (*n* = 79, 6.88 ± 5.80 pg/mL), as illustrated in Figure 2. The minimum and maximum IL-6 values ranged from 4.6 to 38.7 pg/mL in males and 3.7 to 42.8 pg/mL in females. The median IL-6 concentration was 7.15 pg/mL in males (IQR: 5.17–18.86 pg/mL) and 4.96 pg/mL in females (IQR: 4.57–6.45 pg/mL). Given the small number of male participants, a non-parametric test was selected. The Mann–Whitney U test indicated a statistically significant difference between male and female patients (*p* = 0.028), with higher mean ranks observed in males (62.22) compared to females (42.48). This result suggests a possible sex-related difference in IL-6 levels, but the effect size appears modest, and the finding should be carefully interpreted due to the limited number of male patients.

### 3.5. IL-6 Levels and Disease Duration

**Figure 3:**
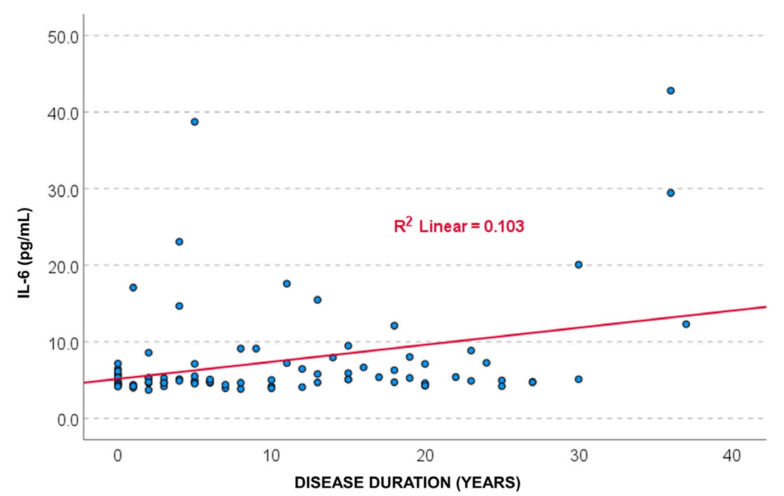
The scatter plot showed a positive association between IL-6 levels and disease duration, with a coefficient of determination R^2^ = 0.103, suggesting that 10.3% of the variance in IL-6 levels is explained by SLE disease duration.

Pearson correlation analysis showed a statistically significant positive association between IL-6 levels and disease duration (r = 0.322, *p* = 0.002). Spearman’s correlation confirmed this result (r = 0.262, *p* = 0.014). These findings suggest a weak directly proportional relationship, indicating that IL-6 concentrations tend to increase slightly with longer disease duration. A simple linear regression model indicated that disease duration significantly predicted IL-6 levels (*p* = 0.002), with a coefficient β = 0.332, 95% CI: 0.082–0.363. The coefficient of determination was R^2^ = 0.103, meaning that disease duration explained only about 10% of the variance in IL-6 levels, which limits the clinical relevance of this association (Figure 3).

### 3.6. IL-6 Levels, Disease Manifestations, and Clinical Manifestations at Presentation

In our cohort of 88 SLE patients, IL-6 levels displayed variable distributions across clinical subgroups, with non-parametric testing (Mann–Whitney U) suggesting possible associations between cytokine elevation and organ involvement (Table 3).

Patients with joint manifestations had higher IL-6 concentrations (8.0 ± 7.4 pg/mL) compared to those without (5.3 ± 1.5 pg/mL), but this difference did not reach statistical significance (*p* = 0.145). Similarly, individuals with cutaneous symptoms exhibited slightly elevated IL-6 levels (8.0 ± 7.8 pg/mL) relative to those without skin involvement (6.5 ± 3.8 pg/mL, *p* = 0.951).

Among patients with mucosal involvement, IL-6 values were also numerically higher (9.5 ± 10.4 pg/mL) than in those without (7.1 ± 5.9 pg/mL), while hematologic manifestations were associated with modestly increased IL-6 levels (7.8 ± 7.4 pg/mL vs. 6.4 ± 3.2 pg/mL, *p* = 0.788). However, none of these comparisons achieved statistical significance.

Serum IL-6 levels were significantly higher in patients with renal involvement (10.6 ± 10.1 pg/mL) compared to those without (6.0 ± 3.6 pg/mL, *p* = 0.032).

Neurological manifestations did not appear to influence IL-6 levels, which were comparable between affected and unaffected patients (7.4 ± 5.8 pg/mL vs. 7.5 ± 7.0 pg/mL, *p* = 0.498).

Interestingly, patients with cardiovascular involvement showed markedly elevated IL-6 concentrations (19.7 ± 20.0 pg/mL) versus those without (7.0 ± 5.6 pg/mL), and this difference was statistically significant (*p* = 0.022). However, this observation is based on only three patients with cardiovascular manifestations. Therefore, these findings require confirmation in larger cohorts.

For pulmonary involvement, IL-6 levels were higher (13.5 ± 16.5 pg/mL) in affected patients than in those without lung manifestations (7.1 ± 5.7 pg/mL), although this difference was not statistically significant (*p* = 0.582). Likewise, IL-6 values were similar in patients with or without serosal involvement (7.3 ± 6.9 pg/mL vs. 7.5 ± 6.7 pg/mL, *p* = 0.242).

In a second step, we performed multivariable binary logistic regression analyses adjusted for age, gender, and disease duration, with the results summarized in Table 4.

Binary logistic regression adjusted for age, sex, and disease duration showed that IL-6 was not significantly associated with articular involvement (OR 0.73; *p* = 0.082). Notably, male gender emerged as an independent predictor of articular manifestations (OR 10.8, *p* = 0.021).

Regression confirmed that higher IL-6 concentrations were independently associated with renal involvement (OR 0.88, 95% CI 0.79–0.99, *p* = 0.026).

The multivariable logistic regression did not confirm significant associations for cardiovascular (OR 0.94, *p* = 0.297), cutaneous (OR 0.96, *p* = 0.376), or hematologic manifestations (OR 0.96, *p* = 0.526).

Logistic regression was not performed for mucosal, serositis, pulmonary, and immunologic involvement, as the low or highly unbalanced number of cases did not allow for a reliable statistical model.

With regard to clinical manifestations at presentation (Table 1), non-parametric comparisons using the Mann–Whitney U test showed that serum IL-6 levels did not differ significantly between patients with and without arthralgia (*p* = 0.687). Similarly, no significant differences were observed for fatigue (*p* = 0.277), cutaneous manifestations (*p* = 0.812), or xerophthalmia/xerostomia (*p* = 0.671).

### 3.7. IL-6 Levels and Disease Activity

**Figure 4:**
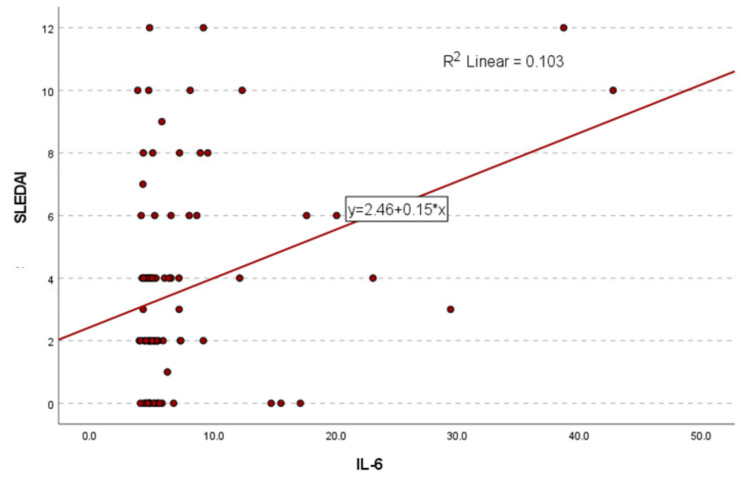
The scatter plot illustrated the relationship between IL-6 levels and SLEDAI total scores. The data points showed a positive trend, supported by the linear regression line and an R^2^ value of 0.103, indicating that IL-6 levels explained approximately 10.3% of the variance in disease activity.

Pearson correlation analysis revealed a statistically significant positive association between IL-6 levels and SLEDAI scores (r = 0.321, *p* = 0.001), indicating a weak to moderate linear relationship. A simple linear regression model further confirmed this association (*p* = 0.002), with IL-6 levels significantly predicting SLEDAI scores. The regression coefficient indicated that for every 1 pg/mL increase in IL-6, the SLEDAI score increased by 0.154 points (β = 0.321, 95% CI: 0.057–0.252). Although the correlation explains only about 10% of the variance in disease activity (R^2^ = 0.103), the finding is clinically relevant, as it supports the role of IL-6 as a potential biomarker of disease activity (Figure 4).

When patients were stratified based on SLEDAI score, mean IL-6 levels were higher in those with active disease (10.8 ± 10.6 pg/mL) compared to patients with inactive disease (6.3 ± 4.4 pg/mL). Median values followed the same direction (7.5 pg/mL vs. 4.9 pg/mL), and the Mann–Whitney U test confirmed a statistically significant difference between the two groups (*p* = 0.025), with a higher mean rank observed in the active subgroup (55.05 vs. 40.98).

**Figure 5:**
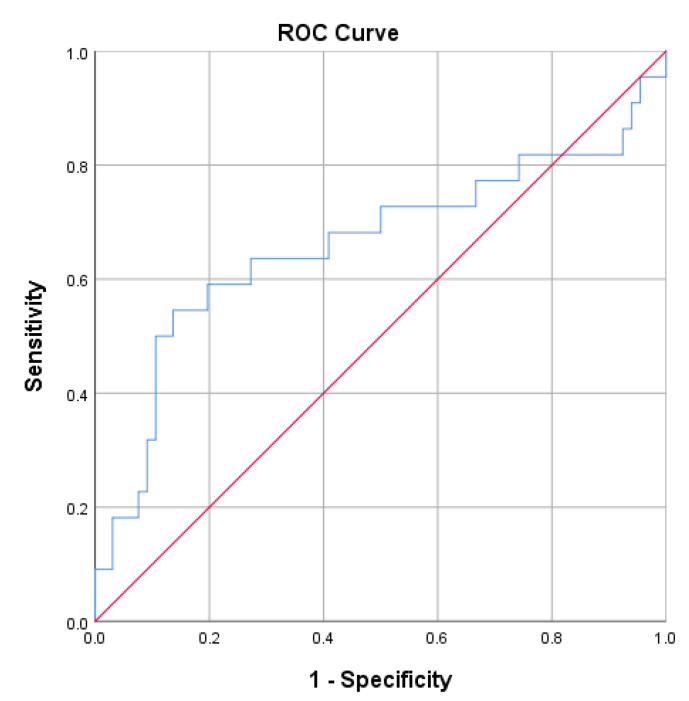
ROC curve analysis for IL-6 in discriminating active (SLEDAI ≥ 6) from inactive disease. The area under the curve (AUC) was 0.66 (95% CI 0.51–0.82; *p* = 0.025), indicating modest discriminatory ability.

ROC analysis further showed that IL-6 had modest discriminatory ability for active disease (SLEDAI ≥ 6) with an AUC of 0,66 (95% CI 0.51–0.82; *p* = 0.025), as presented in Figure 5.

### 3.8. IL-6 Levels, Laboratory Markers, and Treatment

The mean IL-6 serum levels were 7.46 ± 6.73 pg/mL, with values ranging from 3.71 to 42.78 pg/mL. To better understand IL-6’s role in systemic inflammation, we analyzed its possible associations with various laboratory parameters, including hematological, biochemical, and immunological markers and other cytokines (IL-10, IL-17A, and TNF-α), as presented in Table 5.

In our SLE cohort, Spearman’s correlation analysis revealed no significant associations between IL-6 levels and most hematological indices. The correlation with total leukocyte count was weak and non-significant (r = 0.122, *p* = 0.259), and a weak inverse correlation was observed with erythrocyte count (r = –0.164, *p* = 0.127). These effect sizes fall within the weak range, suggesting that IL-6 is unlikely to have a clinically meaningful relationship with these hematological markers in this cohort. Moreover, IL-6 levels also showed no correlation with hemoglobin (r = –0.056, *p* = 0.607), neutrophil count (r = 0.182, *p* = 0.089), or lymphocyte count (r = –0.073, *p* = 0.501).

When patients were divided based on hematological abnormalities, those with lymphopenia showed higher mean IL-6 levels (9.25 ± 10.09 pg/mL) compared to patients without lymphopenia (6.63 ± 4.24 pg/mL), though the difference was not statistically significant (*p* = 0.089). Patients with leukopenia had elevated IL-6 levels (8.23 pg/mL) compared to those without leucopenia (7.22 pg/mL), but this difference was also not statistically significant (*p* = 0.550).

Unlike these hematologic indices, a statistically significant and moderate correlation was observed between IL-6 and ESR, with higher IL-6 levels associated with elevated ESR (r = 0.392, *p* < 0.001). In partial correlation analysis controlling for age, sex, disease duration, and treatment (prednisone, hydroxychloroquine, azathioprine), the association between IL-6 and ESR was no longer significant (r = 0.018, *p* = 0.869), suggesting that the unadjusted correlation may be confounded by these factors (Table 6). No meaningful correlation was found between IL-6 and CRP (r = 0.044, *p* = 0.683).

In our SLE cohort, patients presenting with an active inflammatory syndrome (defined by elevated CRP and/or ESR) exhibited slightly higher IL-6 levels (8.39 ± 7.19 pg/mL) compared to those without inflammatory markers (6.76 ± 6.35 pg/mL). However, this difference did not reach statistical significance (*p* = 0.263).

Significant positive correlations were identified between IL-6 and several biochemical markers. IL-6 levels were moderately associated with serum creatinine (r = 0.378, *p* < 0.001), urea (r = 0.257, *p* = 0.016), and uric acid (r = 0.309, *p* = 0.003), indicating a link between IL-6 and renal function. Moreover, in partial correlation analysis controlling for age, sex, disease duration, and treatment, IL-6 levels remained significantly correlated with serum creatinine (r = 0.246, *p* = 0.026), supporting an independent relationship between IL-6 and renal dysfunction (Table 6).

To further explore the relationship with renal function, patients were stratified based on both biochemical alterations and clinical renal involvement. First, patients were grouped according to the presence or absence of nitrogen retention, defined as elevated serum urea and creatinine levels. Those with nitrogen retention exhibited significantly higher IL-6 levels (10.32 ± 9.16 pg/mL) compared to patients without this alteration (6.57 ± 5.56 pg/mL). This difference was statistically significant (*p* = 0.025).

Additionally, when stratifying patients based on the clinical presence of renal involvement, IL-6 levels were substantially higher among those with renal manifestations. Patients with renal involvement (*n* = 28) had a mean IL-6 serum concentration of 18.15 ± 17.24 pg/mL, compared to 6.95 ± 5.57 pg/mL in those without (*n* = 60). This notable difference was statistically significant, as confirmed by an independent samples *t*-test (*p* = 0.001).

Regarding immunological parameters, Spearman’s analysis indicated that IL-6 was significantly associated with several immunological markers. IL-6 showed a strong positive correlation with ANA (r = 0.435, *p* < 0.001). Additionally, significant associations were found with anti-dsDNA antibodies (r = 0.233, *p* = 0.029), anti-SSA (r = 0.259, *p* = 0.019), and anti-SSB (r = 0.328, *p* = 0.003). These findings provided the rationale for subsequent multivariable logistic regression analyses to further explore the independent associations between IL-6 and specific autoantibody profiles; the corresponding results are presented in Table 7.

Multivariable logistic regression adjusted for age, sex, disease duration, and treatment (corticosteroids, hydroxychloroquine, and azathioprine) showed no significant independent association between IL-6 levels and ANA positivity (OR 0.88, 95% CI 0.72–1.06, *p* = 0.178). None of the covariates were significant predictors, and the overall model was not significant (χ^2^ = 8.8, *p* = 0.264). Partial correlation analysis controlling for the same variables also showed no significant association between IL-6 and ANA titers (r = 0.04, *p* = 0.76).

For anti-dsDNA positivity, IL-6 was not independently associated (OR 1.01, 95% CI 0.94–1.09, *p* = 0.771), and no covariates were significant predictors. The overall model did not reach significance (χ^2^ = 2.2, *p* = 0.949).

Similarly, no significant association was observed between IL-6 and anti-SSA positivity (OR 0.95, 95% CI 0.86–1.05, *p* = 0.343). None of the covariates were significant, and the model was not statistically significant (χ^2^ = 6.8, *p* = 0.448).

For anti-SSB positivity, IL-6 showed a borderline inverse association (OR 0.88, 95% CI 0.77–1.00, *p* = 0.051). The overall model was statistically significant (χ^2^ = 14.3, *p* = 0.045). Corticosteroid treatment showed a non-significant trend towards higher odds of anti-SSB positivity (OR 2.91, 95% CI 0.89–9.52, *p* = 0.077).

When divided into two subgroups, patients positive for anti-SSB antibodies had significantly higher IL-6 concentrations (11.49 ± 11.02 pg/mL) than anti-SSB negative individuals (5.87 ± 3.76 pg/mL). This difference was confirmed by an independent samples *t*-test (*p* = 0.001).

IL-6 levels were examined in relation to complement components. IL-6 was not significantly associated with either complement C3 (r = –0.127, *p* = 0.237) or C4 (r = –0.131, *p* = 0.222).

Using Spearman’s rank correlation, we observed a statistically significant positive correlation between IL-6 and IL-10 (*p* = 0.010). No significant associations were identified between IL-6 and IL-17A (*p* = 0.320) or between IL-6 and TNF-α (*p* = 0.134).

In the analysis of IL-6 concentrations across different therapeutic subgroups (corticosteroids, hydroxychloroquine, azathioprine, methotrexate, mycophenolate mofetil, belimumab, cyclophosphamide, and cyclosporine), no statistically significant associations were observed.

## 4. Discussion

This study offers original insights by analyzing IL-6 in a real-world Eastern European SLE cohort—an underrepresented population in current literature. By assessing IL-6 in relation to disease activity, major organ involvement, renal markers, and autoantibodies such as ANA, anti-SSA, and anti-SSB, we expand on previous findings. Moreover, by including an age- and sex-matched control group, we provide a robust comparative analysis. Notably, elevated IL-6 levels were identified in male patients and in those with renal or cardiovascular involvement, highlighting its potential utility for phenotype-specific biomarker monitoring. These findings support the clinical relevance of IL-6 and warrant further investigation into its diagnostic and therapeutic value in SLE.

Several studies showed that serum IL-6 levels are noticeably higher in SLE patients compared to healthy controls [22,23,37,38,39,40,41,42,43,44,45,46], while other investigations reported no elevation in serum IL-6 levels among SLE patients versus controls [47]. The variability across studies likely reflects differences in disease activity, treatment status, and methodological approaches. Given this heterogeneity in the literature, we analyzed IL-6 concentrations in our cohort. In accordance with most previous reports, mean IL-6 levels were higher in SLE patients than in healthy controls; however, the difference did not reach statistical significance, similar to the findings reported by Mak et al. [48] and Tanaka et al. [49]. The lack of a significant difference in IL-6 levels between SLE patients and controls may be explained by the fact that most patients were receiving immunosuppressive treatment and had low disease activity scores at the time of evaluation, which could have reduced circulating IL-6 concentrations. These considerations suggest that IL-6 may not be considered a diagnostic biomarker distinguishing SLE from healthy individuals but rather a dynamic indicator influenced by treatment and inflammatory activity.

The relationship between IL-6 levels and age in SLE patients has been inconsistently and rarely reported. Our analysis identified a statistically significant but weak positive correlation between IL-6 levels and patient age using Spearman’s method. In line with our findings, the study by Mercader-Salvans et al. [31] on SLE patients reported a positive relationship between age and circulating IL-6 levels. Aging is accompanied by increased production of pro-inflammatory cytokines such as IL-6, contributing to a persistent and low-grade systemic inflammation referred to as “inflammaging” [50]. In the context of SLE, this age-related cytokine increase may overlap with disease-driven inflammation, complicating the interpretation of IL-6 as a biomarker. Our findings showed that, although IL-6 may rise modestly with age, age alone is unlikely to be a strong determinant of IL-6 levels, underscoring the need to account for age-related changes when interpreting cytokine data in SLE cohorts.

We compared serum IL-6 concentrations between male and female SLE patients. The analysis revealed significantly higher IL-6 levels in male patients compared to females. Although the difference was statistically significant, the relatively low proportion of male participants (10.2%) may influence the reliability of this finding. Our results are consistent with those reported by Mercader-Salvans et al. [31], who also observed higher circulating IL-6 levels in male SLE patients, despite men comprising only 8% of their cohort. Several biological mechanisms could underlie this sex-related difference. Proteomic studies showed that male SLE patients have greater neutrophil activation, with higher levels of neutrophil activation markers and reactive oxygen species, which are known to stimulate IL-6 production [51,52]. Additionally, sex hormones may contribute, as male patients have higher follicle-stimulating hormone (FSH) levels, which correlate positively with IL-6 concentrations [53]. Taken together, these findings suggest that sex-specific immunological and hormonal pathways may influence cytokine profiles in SLE. Nevertheless, further studies with larger male cohorts are warranted to validate this observation.

In our cohort of SLE patients, we observed a weak but statistically significant positive correlation between serum IL-6 levels and disease duration, suggesting that individuals with a longer disease course may exhibit higher IL-6 concentrations. This pattern has also been observed in other autoimmune diseases; for example, Tezcan et al. found a significant correlation between IL-6 levels and disease duration in patients with rheumatoid arthritis [54]. However, larger SLE cohorts have not consistently confirmed this association [31]. This discrepancy may reflect differences in sample size and population characteristics and suggests that other factors, such as disease activity, organ involvement, or treatment, should also be considered when interpreting IL-6 levels in SLE.

In our study, involvement of hematologic, immunologic, renal, and other major systems was recorded based on the cumulative diagnostic features of SLE. We found that IL-6 levels were significantly elevated in SLE individuals with renal and cardiovascular involvement. While IL-6 levels appeared higher in other subgroups, such as those with mucosal, articular, or pulmonary manifestations, the differences did not reach statistical significance, possibly due to the small sample size of certain subgroups. The increase in IL-6 levels among patients with cardiovascular involvement aligns with its established role in promoting endothelial dysfunction and atherosclerosis in SLE [55]. Similarly, the significantly higher IL-6 levels observed in patients with renal involvement support previous findings implicating IL-6 in glomerular inflammation and proteinuria [56]. Regarding lupus nephritis, prior studies have demonstrated elevated urinary IL-6 in patients with active renal disease [46,57,58,59,60,61]. Abdel Galil et al. found a significant correlation between IL-6 and active nephritis [62], while Chun et al. reported no clear difference between patients with and without renal involvement [63].

Apart from cardiovascular and renal involvement, associations between IL-6 levels and other organ systems have also been reported. For instance, El-Akhras et al. described a significant association between IL-6 levels and neurological manifestations in SLE [64].

These findings highlight the potential of IL-6 as a potential marker of organ-specific SLE involvement and underscore the need for further validation in larger cohorts.

Considering the link between IL-6 levels and disease activity in SLE, we compared IL-6 concentrations based on SLEDAI scores. Patients with active disease (SLEDAI ≥ 6) showed significantly elevated IL-6 levels compared to those with inactive disease. The positive correlation identified between IL-6 and SLEDAI scores supports the utility of IL-6 as a marker of disease activity, but the modest effect size (explaining only 10.3% of the variance) indicates that additional immunological pathways contribute to disease activity in SLE. This modest yet consistent association across both correlation and regression analyses reflects the multifactorial nature of SLE, involving complex cytokine networks and immune dysregulation. Our findings align with several studies reporting significant correlations between IL-6 levels and disease activity, as assessed by both SLEDAI [23,44,61,62,63,65,66,67,68] and SLEDAI-2K [37,41,42,69,70]. Conversely, other investigations [31,47,48,71] did not demonstrate associations between IL-6 levels and disease activity. Furthermore, a meta-analysis reported an association between IL-6 levels and disease activity only when active disease was defined as SLEDAI > 4, but this relationship was not confirmed when using the SLEDAI-2K > 4 [24]. Elevated IL-6 in active disease may be explained by its role in promoting B-cell differentiation, antibody production, and acute-phase response, mechanisms that become more pronounced during disease flares [23]. In our cohort, the relatively high proportion of patients with inactive disease, many of whom were receiving Disease-Modifying Anti-Rheumatic Drugs (DMARDs), may have further attenuated the strength of the association.

Hematological abnormalities are among the most frequently observed manifestations in SLE patients. Studies report that approximately 63% of individuals with SLE present hematologic involvement, with anemia being the most prevalent [72]. In our cohort, IL-6 levels did not show significant correlations with most hematological indices. This may be partly explained by treatment effects, heterogeneity in disease activity, or the relatively small number of SLE patients within certain hematological subgroups. Previous studies have described an inverse correlation between IL-6 and lymphocyte count, as well as with hemoglobin levels [22,73,74]. Although our findings did not reach statistical significance, IL-6 levels were higher in patients with leukopenia or lymphopenia compared to those without these abnormalities, reflecting trends previously reported in the literature.

Although IL-6 levels were higher in SLE patients presenting with inflammatory syndrome (defined by elevated CRP and/or elevated ESR), the difference did not reach statistical significance. When analyzed separately, IL-6 levels showed a significant positive correlation with ESR. These findings are consistent with previous reports demonstrating a strong association between IL-6 levels and ESR in SLE [63,75,76]. During active SLE, immune activation leads to increased IL-6 production, which stimulates hepatocytes to synthesize acute-phase proteins, including fibrinogen. Elevated fibrinogen, in turn, increases erythrocyte sedimentation rate. This mechanism likely underlies the significant correlation observed between IL-6 and ESR [77,78]. By contrast, no significant correlation was found between IL-6 and CRP levels in our analysis. Nonetheless, the literature includes multiple reports of a positive relationship between IL-6 and CRP [22,31,63,79,80]. It is also important to note that the absence of a correlation between IL-6 and CRP has been previously documented, including in the study by Metsärinne et al. [78,81]. This inconsistency reflects the well-known discrepancy between CRP and other acute-phase reactants in SLE, where CRP often rises less markedly despite ongoing inflammation [78].

Regarding biochemical parameters, the observed associations between IL-6 and renal markers such as creatinine, urea, and uric acid indicate a strong link between elevated IL-6 levels and renal involvement. Serum creatinine and urea are routinely used indicators of renal function, as their accumulation in the blood reflects impaired glomerular filtration and reduced excretory capacity. This is consistent with evidence from acute kidney injury, where systemic IL-6 levels increase in parallel with renal dysfunction and decrease as kidney function improves [82].

In our study, IL-6 levels were significantly associated with ANA and specific autoantibodies, including anti-dsDNA, SSA, and SSB. This aligns with findings by Jin et al. [44], who reported a positive correlation between IL-6 and anti-dsDNA antibodies. However, other studies have not consistently confirmed this association [79,83,84]. One possible explanation is that IL-6 promotes B-cell differentiation and antibody production, thereby contributing to autoantibody generation in SLE. Regarding complement factors, IL-6 levels in our cohort were not significantly correlated with complement components C3 or C4, a result that is consistent with previous reports [84,85].

The significant correlation observed between IL-6 and IL-10 in our cohort aligns with previous studies that have reported similar associations [22,75]. Although we did not find a significant correlation between IL-6 and TNF-α, other investigations documented a positive relationship between these cytokines [61,86].

Several limitations should be acknowledged when interpreting our findings. Serum IL-6 levels were measured at a single time point, limiting the ability to analyze longitudinal changes and their association with treatment or disease flares. In addition, the relatively small number of male participants may have influenced the sex-based differences in IL-6 concentrations. It should also be noted that all patients were under stable immunosuppressive or DMARD therapy at the time of blood collection, which may have influenced serum IL-6 levels. The predominantly inactive disease status of our cohort could have led to an underestimation of the relationship between IL-6 levels and disease activity. Moreover, healthy controls were not systematically screened for all SLE-associated autoantibodies, which may represent a potential source of bias, despite the absence of autoimmune symptoms.

Future research should address these limitations through prospective longitudinal studies, including serial IL-6 measurements and stratification by treatment phase and flare status. Broader cohorts with greater representation of male patients and patients with active disease are also needed.

## 5. Conclusions

Our findings reinforce the involvement of IL-6 in multiple immunological and clinical aspects of SLE. IL-6 levels in SLE were associated with disease activity, renal dysfunction, and immunological markers. Concentrations were higher in patients with active disease and renal involvement, and additional associations were observed with IL-10 and anti-SSB antibodies. These findings underscore the biological relevance of IL-6 in SLE and indicate that its clinical utility is more appropriately considered within a composite panel of biomarkers rather than as an independent indicator. Future studies should focus on longitudinal assessments and larger, more diverse cohorts to clarify the clinical utility of IL-6 in disease monitoring.

## Figures and Tables

**Table 1:** Baseline demographic and clinical characteristics of SLE patients and comparison between SLE patients and healthy controls.

Parameter	Patients with SLE (*n* = 88)/Number (%)	Controls (*n* = 87)/ Number (%)
Female/Male	79/9 (89.8/10.2%)	78/9 (89.7/10.3%)
Age	51.17 ± 15.36	51.38 ± 15.30
Disease duration	10.38 ± 9.72	
Clinical features		
Arthralgia	70/88 (79.6%)	
Fatigue	25/88 (28.4%)	
Cutaneous manifestations	19/88 (21.6%)	
Xerophthalmia/Xerostomia	18/88 (20.5%)	
Disease activity (SLEDAI)		
SLEDAI ≥ 6	22/88 (25%)	
SLEDAI < 6	66/88 (75%)	
Treatment		
Hydroxychloroquine	79/88 (89.8%)	
Azathioprine	30/88 (34.1%)	
Methotrexate	7/88 (8%)	
Mycophenolate mofetil	4/88 (4.5%)	
Belimumab	8/88 (9.1%)	
Corticosteroids	33/88 (37.5%)	

**Table 2:** Comparison of serum IL-6 levels between SLE patients and healthy controls.

Subjects	Number	Mean ± SD (pg/mL)	Min–Max (pg/mL)
SLE Patients	88	7.46 ± 6.73	3.71–42.78
Controls	87	5.31 ± 10.9	1.85–101.33

**Table 3:** IL-6 levels across different SLE manifestations.

Disease Manifestation	Percentage/Number of Patients	*p*-Value (Mann–Whitney U Test)
Articular Involvement	79.5% (70/88)	0.145
Cutaneous Involvement	65.9% (58/88)	0.951
Mucosal Involvement	14.8% (13/88)	0.362
Hematologic Involvement	78.4% (69/88)	0.788
Immunologic Involvement	97.7% (86/88)	0.576
Serositis	17% (15/88)	0.242
Renal Involvement	31.8% (28/88)	0.032
Neuropsychiatric Involvement	22.7% (20/88)	0.498
Cardiovascular Involvement	3.4% (3/88)	0.022
Pulmonary Involvement	5.7% (5/88)	0.582

**Table 4:** Multivariable logistic regression analyses for associations between serum IL-6 levels and major clinical manifestations in SLE.

Outcome (Dependent Variable)	*n* (Cases)	OR for IL-6 (95% CI)	*p*-Value (IL-6)	Other Significant Covariates
Renal involvement	28	0.88 (0.79–0.99)	0.026	None
Cardiovascular involvement	3	0.94 (0.84–1.06)	0.297	None
Articular involvement	70	0.73 (0.52–1.04)	0.082	Gender: OR 10.80, *p* = 0.021
Cutaneous involvement	58	0.96 (0.87–1.05)	0.376	None
Hematologic involvement	69	0.96 (0.86–1.08)	0.526	None

Adjusted for age, sex, and disease duration.

**Table 5:** Correlations between IL-6 levels and hematological, biochemical, and immunological markers.

Laboratory Parameters	Spearman’s Rank Correlation Coefficient (R)	*p*-Value
Hematological Parameters		
RBC	−0.164	0.127
HGB	−0.056	0.607
HCT	−0.035	0.747
PLT	0.181	0.092
WBC	0.122	0.259
NEU	0.182	0.089
LYMPH	−0.073	0.501
Biochemical Parameters		
ESR	0.392	<0.001
CRP	0.044	0.683
UREA	0.257	0.016
CREAT	0.378	<0.001
UA	0.309	0.003
Immunological Parameters		
ANA	0.435	<0.001
Anti-dsDNA	0.233	0.029
SSA	0.259	0.019
SSB	0.328	0.003
RNP-70	0.132	0.348
C3	−0.127	0.237
C4	0.049	0.800
Pro-inflammatory cytokines		
IL-10	0.274	0.010
IL-17A	0.107	0.32
TNF-α	0.134	0.134

RBC = Red Blood Cells; HGB = Hemoglobin; HCT = Hematocrit; PLT = Platelets; WBC = White Blood Cells; NEU = Neutrophils; LYMPH = Lymphocytes; CREAT = Creatinine; UA = Uric Acid; ESR = Erythrocyte Sedimentation Rate; CRP = C-Reactive Protein; SI = Serum Iron; ANA = Antinuclear Antibodies; Anti-dsDNA = Anti-Double-Stranded DNA Antibodies; SSA = Anti-SSA Antibodies; SSB = Anti-SSB Antibodies; C3 = Complement C3; C4 = Complement C4; IL-6 = Interleukin-6; IL-17A = Interleukin-17A; TNF-α = Tumor Necrosis Factor Alpha.

**Table 6:** Partial correlations between cytokine levels and laboratory markers after adjustment for age, sex, disease duration, and treatment.

Correlation Pair	r	*p*-Value
IL-6 vs. ESR	0.018	0.869
IL-6 vs. Creatinine	0.246	0.026

Adjusted for age, sex, disease duration, prednisone, hydroxychloroquine, and azathioprine.

**Table 7:** Multivariable logistic regression analyses for associations between serum IL-6 levels and autoantibody profiles in SLE.

Outcome (Dependent Variable)	OR for IL-6 (95% CI)	*p*-Value (IL-6)	Other Significant Covariates
ANA positivity	0.88 (0.72–1.06)	0.178	None
Anti-dsDNA positivity	1.01 (0.94–1.09)	0.771	None
Anti-SSA positivity	0.95 (0.86–1.05)	0.343	None
Anti-SSB positivity	0.88 (0.77–1.00)	0.051	Corticosteroids (OR 2.91, *p* = 0.077)

Adjusted for age, sex, disease duration, and treatment (corticosteroids, hydroxychloroquine, and azathioprine).

## Data Availability

The original contributions presented in this study are included within this article. Further inquiries can be directed to the corresponding authors.
